# DNA Aptamer Functionalized Hydrogels for Interferometric Fiber-Optic Based Continuous Monitoring of Potassium Ions

**DOI:** 10.3390/bios11080266

**Published:** 2021-08-06

**Authors:** Nataša Žuržul, Bjørn Torger Stokke

**Affiliations:** Biophysics and Medical Technology, Department of Physics, NTNU The Norwegian University of Science and Technology, NO-7491 Trondheim, Norway; natasa.zurzul@ntnu.no

**Keywords:** potassium biosensor, aptamer, responsive hydrogel, interferometry

## Abstract

In the present paper, we describe a potassium sensor based on DNA-aptamer functionalized hydrogel, that is capable of continuous label-free potassium ion (K^+^) monitoring with potential for in situ application. A hydrogel attached to the end of an optical fiber is designed with di-oligonucleotides grafted to the polymer network that may serve as network junctions in addition to the covalent crosslinks. Specific affinity toward K^+^ is based on exploiting a particular aptamer that exhibits conformational transition from single-stranded DNA to G-quadruplex formed by the di-oligonucleotide in the presence of K^+^. Integration of this aptamer into the hydrogel transforms the K^+^ specific conformational transition to a K^+^ concentration dependent deswelling of the hydrogel. High-resolution interferometry monitors changes in extent of swelling at 1 Hz and 2 nm resolution for the hydrogel matrix of 50 µm. The developed hydrogel-based biosensor displayed high selectivity for K^+^ ions in the concentration range up to 10 mM, in the presence of physiological concentrations of Na^+^. Additionally, the concentration dependent and selective K^+^ detection demonstrated in the artificial blood buffer environment, both at room and physiological temperatures, suggests substantial potential for practical applications such as monitoring of potassium ion concentration in blood levels in intensive care medicine.

## 1. Introduction

Potassium is one of the main electrolytes and the second most abundant type of cation present in the human body. It plays an important role in many physiological functions, including cellular metabolism, glycogen and protein synthesis, and regulation of electrical action potential across cell membranes, and therefore is essential for normal functioning of the cardiovascular system, skeletal muscle, internal organs, and nervous system. Potassium disorders occur in up to nearly one third (31%) of hospitalized patients [1]. Hypokalemia and hyperkalemia are common electrolyte disorders caused by down- and up-regulated potassium levels. Among the most common causes of hypokalemia, diuretic use and gastrointestinal losses are prevailing, whereas kidney disease, diabetes and medication use are the most common cause of hyperkalemia. When severe, potassium disorders can lead to life-threatening cardiac rhythm disturbances and neuromuscular dysfunction which may provoke sudden cardiac arrest or respiratory failure. Critically ill patients with potassium disorders had a higher incidence of cardiac arrhythmia and intensive care unit (ICU) mortality than patients with normal potassium levels [2,3,4,5,6,7,8,9,10,11,12]. Therefore, an effective, rapid, and selective method for continuous point-of-care (POC) potassium monitoring is highly desirable, particularly in the ICU where timely therapy is of crucial importance. 

Despite efforts, it is still difficult to selectively determine K^+^ concentrations, due to the presence of other ions, especially the large excess of Na^+^ in biological fluids. Furthermore, most of the K^+^ assays require frequent sample collection. Frequent blood sampling is not desired due to anemic conditions in some patients. The potassium levels can be determined from blood, urine, sweat, saliva, or tears [13]. The most accurate method for evaluating urinary potassium excretion is a 24-h timed urine potassium collection; however this method is too long for providing optimal treatment promptly. The sweat, saliva, or tears collection are challenging due to evaporation, the requirement for stimulus, and differing secretion rates and compositions among individuals and daily conditions [14,15]. Quantitative methods for measuring K^+^ levels in patients admitted to ICU are from blood, conventionally using benchtop auto-analyzers (AA), measurements based on ion-selective electrodes, or by gas analysis using arterial blood gas (ABG) analyzers [16,17,18]. Such measurement strategies require frequent manual blood sample collections. AA require transportation of the sample to a clinical biochemistry laboratory and sample testing which results in relatively long analysis times, while ABG analyzers more readily provide results but require highly skilled personnel for sampling blood from an artery. Serum K^+^ levels should be monitored frequently in patients with potassium disorders if symptoms are present, when treatment is ongoing, and after the treatment. Routine daily monitoring is also recommended for adult patients admitted to ICU. Although current techniques are satisfactory in many situations, life-threatening episodes of potassium disturbance might often not be detected by such an intermittent technique. Moreover, aside from being inconvenient, frequent blood sampling is not desired due to anemic conditions in some patients [6,19,20,21].

Hydrogel properties including hydrophilic character, elastic nature, biocompatibility, and versatility to tailor-make physical and chemical properties by incorporation of different moieties, contribute to their high potential for application in bioanalytical fields and biosensing [22,23,24,25,26,27,28,29]. Aptamer-based hydrogels exploiting the unique properties of functional oligonucleotide chain motifs, such as specific molecular recognition, programmable and high-precision assembly, and multifunctionality have attracted increasing research interest in biosensor fabrication [30,31,32,33]. Aptamers often possess very high affinity and selectivity for their targets. This originates from the nucleotide chains folding upon binding with their target molecule by either incorporating small molecules into their structure or being integrated into the structure of macromolecules [34]. Furthermore, miniaturization of aptamer-based hydrogels to the micro- and nanoscale leads to better responsiveness, thereby making them excellent candidates for in vivo real-time sensing. Biomolecule recognition within aptamers integrated with hydrogels may induce responses by changing the hydrogel swelling volume, crosslinking density, and optical or mechanical properties, which enables monitoring by using the appropriate readout platform, thus exploiting the responsive hydrogel as the recognition and transducing functionality in a sensor [35,36,37,38,39,40].

Previously reported aptamer-based potassium biosensors designed with fluorescence readout for sample testing [41,42] are not a preferred design for continuous potassium monitoring. Apart from aptamer-based potassium biosensors, various other K^+^ sensing approaches have been reported, such as ones based on selective binding to crown ether functionalized polymers or hydrogels integrated in quartz crystal microbalance for readout [43,44], or a similar moiety integrated in self-assembled monolayers on an Au surface for electrochemical readout [45]. These studies reported selectivity of K^+^ determination based on the complex formation between the crown ethers and tight interaction with this alkali metal ion, in the concentration range 0.5–7 mM K^+^. The selective K^+^ monitoring using graphene-based ion sensitive field effect transistors exploits valinomycin in the membrane coating to mediate K^+^ selectivity [46]. The latter strategy has also extended to measurements in the presence of physiological levels of other ions. Moreover, a paper-based microfluidic approach resulting in K^+^ dependent analytical readout as the migration distance of a colored substance has recently been reported [47].

In the present study, we explore K^+^ aptamer-functionalized hydrogels as a specific recognition and transducing element, that combined with a fiber optic based high resolution readout of the hydrogel swelling, constitute a functional biosensor. Fiber-optic based readout platforms that have been widely explored in chemical and biological sensing (e.g., [48,49]), including also determination of selected ions [50,51], offer various advantages; in particular, not being prone to electromagnetic interference. Compared to the traditional quantitative methods for K^+^ sampling in ICU patients, the presented potassium biosensor does not face impediments such as frequent blood sample collection, complicated sample preparation, long assay time, or requirement for highly trained personnel. It is capable of continuous potassium monitoring and non-invasive implementation. Its miniaturized size as integrated on the optical fiber allows incorporation or direct insertion into an indwelling arterial catheter without disrupting that catheter’s current use, resulting in fast and simple handling.

## 2. Materials and Methods

### 2.1. Materials

The following materials were obtained from Sigma-Aldrich: acrylamide (99%, AAm), N,N’-methylenebisacrylamide (99%, Bis), 1-hydroxycyclohexyl phenyl ketone (99%, HCPK), squalene (96%), 3-(trimethoxysilyl)propyl methacrylate (98%), dimethyl sulfoxide (DMSO), Tris base (≥99%, Trizma base), Tris hydrochloride (≥99%, Trizma HCl), sodium phosphate monobasic monohydrate (≥99%, NaH_2_PO_4_ · H_2_O), sodium phosphate dibasic (≥99%, Na_2_HPO_4_), sodium chloride (99.5%, NaCl), potassium chloride (99%, KCl), calcium chloride (97%, CaCl_2_), magnesium chloride (98%, MgCl_2_), bovine serum albumin (96%, BSA), HCl (37%), ethanol (used for cleaning only). Ultrapure water, resistivity 18.2 MΩ cm (Milli-Q plus, Merck Millipore, Burlington, MA, USA), was used throughout the experiments. All the oligonucleotides were obtained from Integrated DNA Technologies (IDT, Coralville, USA). The sequences of the oligonucleotides were custom designed (Table 1), selected based on the reported potassium aptamer sequences for the oligo-3 [42] and KLM [52] aptamers, 5′-TGAGGGAGGGG-3′ and 5′- GGGTTAGGGTTAGGGTTAGGG-3′, respectively. The core aptamer sequences are extended by 4 bp at the end to allow for flexible anchoring of the nucleotide aptamers to the acrylamide network. Acrydite functionalities at the end of the aptamers support their covalent integration in the AAm network [53,54].

### 2.2. Sensor Fabrication

Aptamer functionalized hydrogels were fabricated covalently attached at the end of optical fiber, supporting determination of changes in the optical lengths at high resolution. Acrylamide (AAm) (10 wt%) pre-gel solutions were prepared with Bis (0.6 mol% relative to AAm) as the crosslinker. Oligonucleotides (0.4 mol% relative to AAm) were included in the pre-gel solution. The polymerization was initiated by the photoinitiator (HCPK) included in the pre-gel solution at a concentration 0.13 mol% relative to the AAm monomer. The Tris-HCl buffer (50 mM, pH 7.4) was prepared with addition of KCl and used as a solvent for oligo-3 pre-gel solution. The pre-gel solutions including the KLM-L and KLMss aptamers are prepared by dissolving the chemicals in the same concentration ratio in Milli-Q water.

A small aliquot of the pre-gel solution was deposited using a pipette at the end of the functionalized optical fiber immersed in a hanging droplet of squalene oil, while inspected through a stereo microscope. The optical fibers were functionalized following a previous protocol [55]. In brief, this includes the following steps. The optical fibers were first stripped, cut (Fitel model S323, Furukawa Electric Co. Ltd. Tokyo, Japan), and cleaned with ethanol, water, and tape for removing possible particular contaminants. The silanization proceeded by immersing the optical fiber tip in 100 mM NaCl solution (15 min), cleaning with ethanol and water, and subsequently immersing in 2 mol% silane solution (15 min) to chemically bind the silane terminated with methacrylate groups. The silane solution is prepared by making 0.02 M solution of 3-(trimethoxysilyl)propyl methacrylate in nitrogen purged Milli-Q water and adjusted to pH 3.5.

The pre-gel at the end of the silanized optical fibre in squalene droplet (containing 2.6 mg/mL of the photoinitiator) forms a hemispherical droplet due to the surface tension. The polymerization is initiated by exposure to UV light (Thorlabs T-Cube LED Driver) for 300 s. Following polymerization, the gel is washed by leaving it in Tris-HCl buffer without KCl for at least 12 h to eliminate K^+^ ions, unreacted monomers, and other impurities.

Hydrogels containing various potassium aptamers were preliminary tested in 0.1 M sodium phosphate buffer (pH 7.4) for K^+^ and Na^+^ ion selectivity, since Na^+^ is the most abundant ion in human blood. Due to the ability of phosphates to form insoluble salts with bivalent metals, such as Ca^2+^ and Mg^2+^, and precipitate, Tris-HCl buffer (50 mM, pH 7.4, 140 mM NaCl) was used instead of phosphate buffer for selectivity testing [56,57]. 

### 2.3. Determination of Biosensor Swelling Response

The aptamer-co-AAm hydrogel-based biosensors were characterized with respect to changes in optical length of the hydrogel in aqueous buffer solutions, with emphasis on potassium sensitivity and selectivity. This was also extended to artificial blood buffer as a first indication of a possible direct potassium determination, e.g., to test possible interference with other components in the liquid biopsy to be analyzed. The fibers with the hydrogels at the end were immersed into an appropriate volume of aqueous Tris-HCl buffer (50 mM, pH 7.4) with 140 mM NaCl, corresponding to the average physiological level of NaCl in human serum. The changes in the optical length associated with the stepwise increase in concentration of selected salts were assessed by consecutively adding aliquots of 0.1 M stock solutions of KCl, NaCl, CaCl_2_, and MgCl_2_ to the aqueous Tris-HCl buffer. The responses to KCl and NaCl were determined for concentrations up to 10 mM added to the initial buffer concentrations, whereas changes in swelling response were determined for CaCl_2_ and MgCl_2_ to the upper value of the normal level in human serum, 2.5 mM and 1 mM, respectively. Subsequent steps in the increase of salt concentrations were introduced once the equilibrium was reached, manifested by a constant phase of the interference wave of the readout platform. Following completion of each series of determination of effects of increasing salt in the immersing solution, the hydrogels were washed and left in Tris-HCl buffer (50 mM, pH 7.4, 140 mM NaCl) before repeating the gel swelling experiments. Experiments were carried out at room temperature with the fiber tip containing polymerized gel immersed in the buffer solution under constant agitation, using a magnetic stirrer at speed 100 min^−1^.

The experiments in artificial blood buffer were conducted by stepwise addition of aqueous KCl stock solution up to 10 mM. This series of experiments were conducted both at room temperature and at the temperature of the human body (37 °C). The required operating range of the sensor to measure deviations from the normal K^+^ values relevant for clinical practice is tested in this series of experiments [58]. Artificial blood buffer is made by adding average physiological values of the salts and protein (Table 2) to the Tris-HCl buffer (50 mM, pH 7.4) [59,60]. All experiments were repeated at least twice.

The functionalized hydrogels at the optical fibers were characterized with respect to changes in swelling response, using an interferometric readout platform providing data on changes of 2 nm resolution in the optical length for the nearly hemispherical hydrogel of 50 µm radius [55]. The complete set-up including hardware and LabView program for data collection was obtained from Invivosense ASA, Trondheim, Norway. The interferometric determination of the optical length in the hydrogel exploits a tunable super fluorescent Er-doped optical fiber source (wave-packet λ_0_ = λ_a_ − λ_b_ = 1530–1560 nm) [61]. This IR signal used as the incident light is partly reflected at the fiber-hydrogel (reflection coefficient *r*_1_) and hydrogel-immersing solution interfaces (reflection coefficient *r*_2_), and a net reflected interference wave with amplitude and phase that change depending on the swelling of the hydrogel:

(1) $$I\left(\lambda \right)\approx I_{0}\left(r_{1}^{2}+\gamma^{2}r_{2}^{2}+2\gamma r_{1}r_{2}cos\left(4\pi l_{o}/\lambda \right)\right)$$

where *I*_0_ is the incident intensity, *l*_o_ is the optical length of the gel, and γ is the loss factor. The change in the phase is found to yield the highest resolution data of the optical length,

(2) Δθ=θ(Δt)−θ(0)

(3) $$\Delta l_{o}=\frac{\Delta \theta \;\lambda_{0}}{4\pi }$$

where λ_0_ is the center wavelength of the source spectrum, Δ*θ* is the phase change from the start of the measurement to the time Δ*t*. The interferometric set-up is controlled by a computer and a LabView program used for instrument readout. The sampling rate is about 1 Hz, supporting determination of kinetic aspects of the hydrogel swelling as well. This setup enables high accuracy detection of small changes in stimuli-responsive hydrogels and substantially faster readout than methods exploiting larger sizes of the hydrogel specimen [55]. The standard deviations of the relative change of the optical length, Δ*l*_o_/*l*_o_, for the equilibrium data were calculated from the ten last data points prior to changing salt concentration in the immersing bath. The recorded changes in the optical length are in most cases found to be dominated by changes in the physical length along the optical axes, and the possible concomitant change in the refractive index associated with changes in swelling state are much less pronounced.

## 3. Results and Discussion

**Figure 1:**
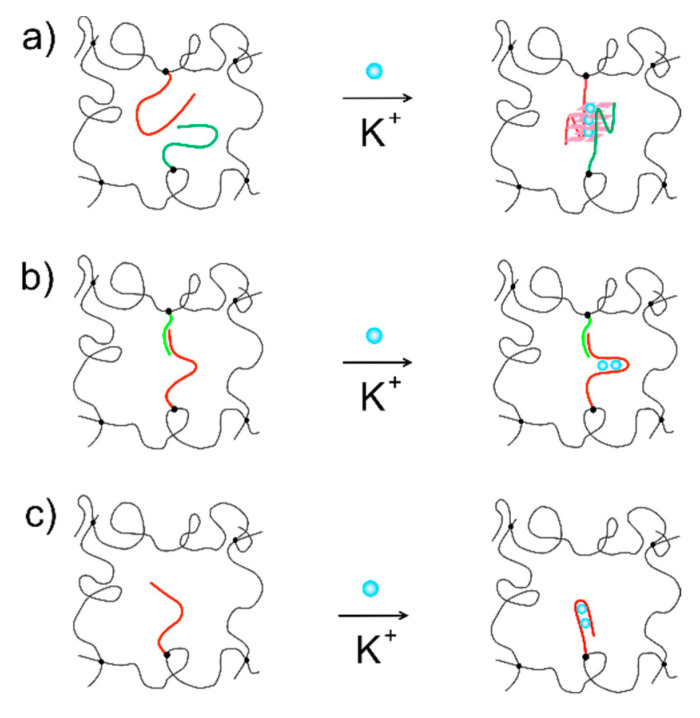
Schematic illustration of potassium aptamer sequence structures and their integration in hydrogels for label-free biosensing. (**a**) Oligo-3; (**b**) KLM-L; (**c**) KLMss.

Three different K^+^ aptamers (Table 1) integrated in acrylamide hydrogels (Figure 1) were prepared as sensing elements at the end of the optical fiber. These different types of aptamer-co-AAm hybrid hydrogels were assessed with respect to specificity and sensitivity in inducing changes in their swelling state. Focus is on response to K^+^ in the aqueous immersing solution, and thereby their feasibility as a specific recognition and transducing element. The KLM linked with L, thus forming an additional crosslink, and KLMss aptamers were incorporated as single stranded grafts to the network structure, whereas the oligo-3 aptamer requiring two identical molecules for formation of the highly K^+^ selective complex was included as a paired duplex. Additionally, we also included an oligonucleotide with random bp sequence as a control.

In the absence of K^+^, the nucleotides reported as potassium aptamers adopt a ssDNA conformation, e.g., a quite flexible structure with a persistence length in the order of 1 nm in 0.15 M aqueous salt solutions [62]. The actual bp composition and sequence may influence the actual chain stiffness [63]. Addition of K^+^ leads to formation of K^+^-aptamer specific interactions, resulting in formation of favorable geometry for the interaction. Specifically, a G-quadruplex structure (Figure 1) is formed in the case of the K^+^-induced structure of the two oligo-3 aptamers.

**Figure 2:**
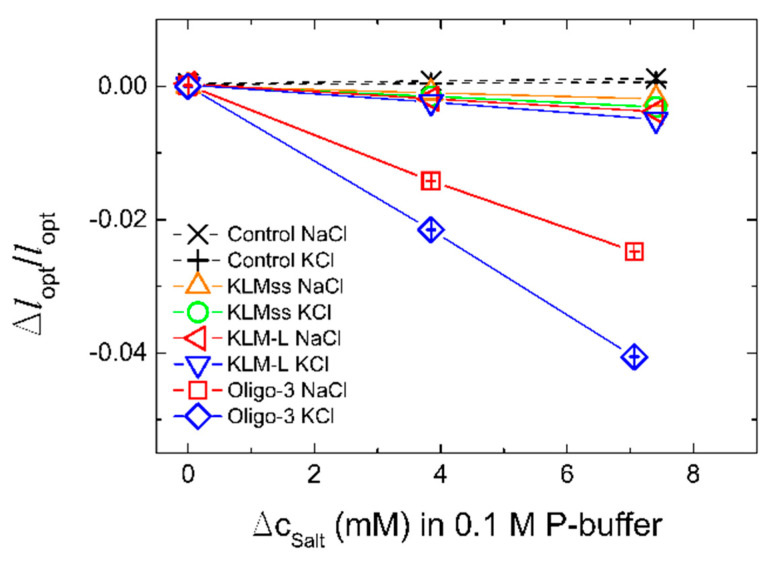
Sensitivity and selectivity of biosensor hydrogels incorporating either oligo-3, KLM-L, or KLMss oligonucleotides as a function of the K^+^ and Na^+^ ions, added to the 0.1 M phosphate buffer (pH 7.4) in two steps. The sensitivity was determined as the relative change in optical length of the hydrogels when they are changing their extent of swelling as function of salt concentration for Na^+^ and K^+^. Acrylamide gel with corresponding concentrations of acrylamide and numbers of crosslinks, without incorporated DNA sequences, is used as the control. The standard deviation of Δ*l*_opt_/*l*_opt_ for the oligo-3-co-AAm hydrogel sensor are shown at 0, 3.8 and 7.1 mM of NaCl and KCl added to the phosphate buffer.

In the initial experiments, we compared potassium-induced changes of the optical length of the hydrogels with the various aptamer oligonucleotide sequences. The potassium-induced changes were compared to those in the presence of added sodium, for concentrations up to 7 mM in an aqueous solution of 0.1 M phosphate buffer (note that this buffer condition implies a sodium concentration of 0.177 M originating from the phosphates used for the buffer preparation). The experimental data indicate KLM linked with L and KLMss were showing lower selectivity between potassium and additional sodium ions compared to the hydrogel with the integrated oligo-3 aptamer (Figure 2). The finding of no relative change in the optical lengths of the pure acrylamide hydrogels, used as the control in this case, indicate that the responses observed for the hydrogels with the various aptamers are due to these entities.

**Figure 3:**
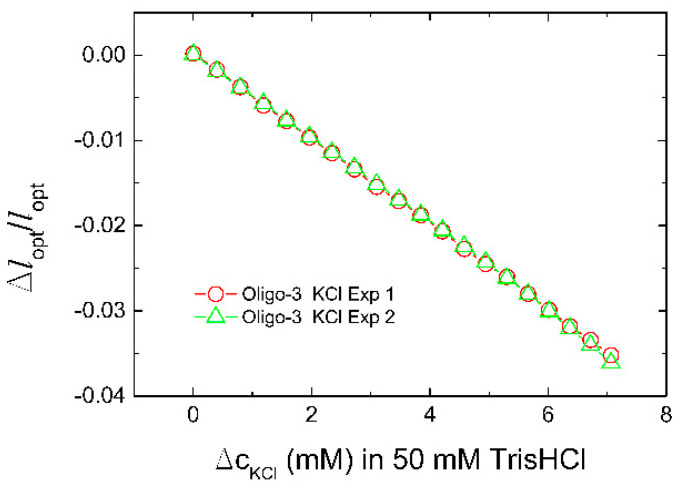
Repeated experimental determination of change of relative optical length versus concentration of KCl added to 50 mM Tris buffer (pH 7.4) for oligo-3-co-AAm hydrogel sensing element at the end of an optical fiber. The hydrogel sensor was extensively washed in 50 mM Tris buffer (pH 7.4) following completion of the first experimental series (Exp 1) to re-equilibrate the hydrogel, followed by step-wise increase of KCl in the second experimental series (Exp 2).

**Figure 4:**
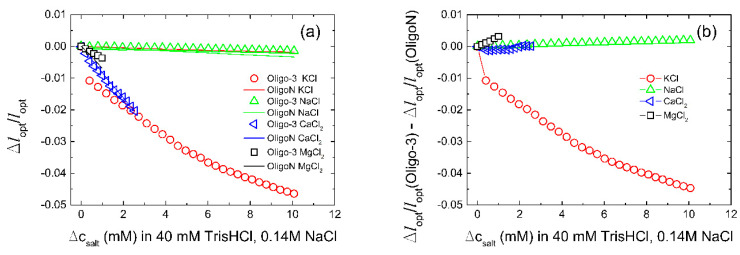
Sensor selectivity. (**a**) Change in the optical length of the sensor and control gels as a function of K^+^, Na^+^, Ca^2^^+^, Mg^2^^+^ ions added to at least upper physiological level concentration. The experiments were carried out in Tris-HCl buffer (50 mM, pH 7.4, 140 mM NaCl) at room temperature. (**b**) Selectivity of the oligo-3 biosensor relative to random oligoN-co-AAm hydrogel sensors for the various salts KCl, NaCl, CaCl_2_, and MgCl_2_. The selectivity is estimated as the difference in the relative change of the optical lengths versus salt concentrations for the oligo-3-co-AAm and random oligoN-co-AAm.

The standard deviations of the relative change of the optical length Δ*l*_opt_/*l*_opt_ in the case of oligo-3-co-AAm sensor determined at increasing NaCl and KCl to the buffer (Figure 2) were observed to be of the order up to 5 × 10^−5^. The graphical presentations of these uncertainties were found to be included within the size of the symbols used (Figure 2), and also in the case of all equilibrated data points (Figure 2, Figure 3 and Figure 4 and Figure 6). Repeated exposure of the oligo-3-co-AAm hydrogel to increasing KCl in the immersing aqueous solution shows similar deswelling behavior (Figure 3). In this experiment, the hydrogel sensor was washed in buffer (50 mM Tris, pH 7.4, without KCl) after completion of the first series. This indicates that the K-induced changes in this oligo-3-co-AAm hydrogel are reversible.

The inclusion of the aptamers in the uncharged hydrogel change the hydrogel from being neutral to an anionic hydrogel. In addition, oligo-3 and KLM-L are introduced with a topology that may affect the length of aptamer associated with the interaction with the potassium. The association constants of potassium and sodium for the KLMss aptamer are reported to be K_a_ = (1.3 ± 0.2) × 10^7^ and (3.0 ± 0) × 10^2^ M^−2^ for KCl and NaCl (for 0.21 µM of aptamer in aqueous 5 mM Tris-HCl at 25 °C), respectively [64]. These association constants were obtained assuming a model where two cations were required to bind to each aptamer, thus yielding the units as stated. Applying these values for the current hydrogel exposed to the aqueous NaCl and KCl solutions, we find that the KLMss aptamers should be completely saturated with K^+^ for KCl concentrations above 2 mM, whereas only less than 1% of the KLMss aptamers binds Na^+^ in the same range. Although there is a marginal larger response in the presence of KCl as compared to NaCl, the hydrogels with the KLMss aptamer appear not to transform the reported selectivity between Na^+^ and K^+^ to similar selective changes in swelling response of AAm grafted with KLMss aptamers. The experimental data show a larger sensitivity for Na^+^ and K^+^ in the case of the oligo-3 aptamer, with the largest deswelling effect in the presence of K^+^ as compared to Na^+^ (Figure 2). Thus, in the following, we proceed with more in-depth proof-of-concept validation of the oligo-3 aptamer integrated in the hydrogel as a K^+^-selective recognition and transducing element.

The K^+^-induced swelling of hydrogels with the oligo-3 aptamer was compared to that of hydrogels where the oligo-3 aptamer had been changed to a oligonucleotide with random bp sequence oligoN, e.g., sequences that are not specific for recognition of K^+^ (control sequences). Such comparison aids in the assessment of the specific effect of K^+^, and should be included since introduction of the aptamer in the AAm gels change the material from being an uncharged hydrogel to an anionic hydrogel responding to changes in salt concentration. Although the specificity tests conducted here are performed by addition of the various ions to a solution with considerable ionic strength, the tests are included to assess the specificity. Among the monovalent ions, the data (Figure 4) show that the oligo-3-co-AAm hydrogel deswells with increasing KCl concentration up to 10 mM in the aqueous immersing bath with 150 mM salt, and buffer. The extent of deswelling in the linear dimension of the hydrogel is −4.5% for K^+^ at 10 mM, relative to 0 mM.

This contrasts the lack of response of the same hydrogel when increasing NaCl up to 10 mM in the same reference solution, clearly indicating that the potassium aptamer functionality of the oligo-3 duplex is selective to K^+^ relative to Na^+^, and that the K^+^-induced oligo-3 duplex conformational rearrangement can be transduced to a hydrogel deswelling. It is further observed that the oligo-3-co-AAm hydrogel deswells in the presence of added divalent cation Ca and Mg salts to the aqueous buffer salt solution, tested up to the physiological range (Table 2). However, it should be noted that a similar deswelling is also observed for the oligoN-co-AAm gel (Figure 4b), indicating it is not specific. The rather small deswelling of the oligoN-co-AAm on increasing concentrations of monovalent ions (KCl and NaCl), and the more substantial deswelling of this hydrogel sensing element on increasing concentrations of divalent ions (CaCl_2_ and MgCl_2_), are generally considered to be consistent with the deswelling response of ionic hydrogels [65]. The selectivity between the oligo-3-co-AAm relative to the oligo-N-co-AAm hydrogel sensing elements with respect to the net transducing effect (Figure 4b) shows that it is only the KCl that is yielding the ion specific difference. This KCl specific selectivity is persisting to higher KCl concentration (Figure 4b), indicating that further exploration as a biosensor for hypo/hyperkalemia should be conducted.

**Figure 5:**
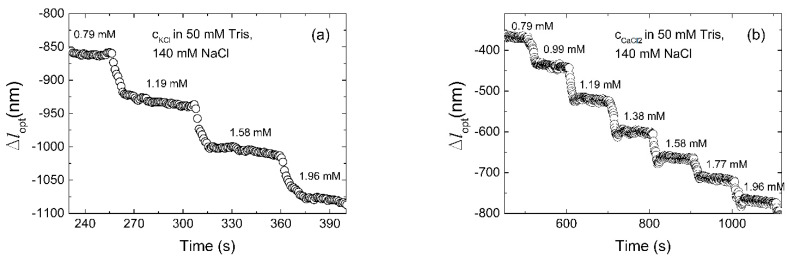
Optical length of the sensor versus time for stepwise 0.794–1.962 mM increase in (**a**) KCl and (**b**) CaCl_2_. Tran-sient swelling when K+ ions are added is slower as a result of the influence of the kinetics of folding of the aptamer, compared to Ca^2+^ ions addition.

The rate of deswelling to the new equilibrium state for stepwise increase in K^+^ and Ca^2+^ (Figure 5) shows that the process apparently can be considered as a two-step process, where, in particular, the first re-equilibration reached within 10 s is followed by a slower process. In the case of deswelling of the oligo-3-co-AAm occurring for a step-change in K^+^, it could be understood in view of kinetics of 3D folding of the aptamer into the G-quadruplex, combined with the network relaxation. The latter process is dependent on the size of the hydrogel, and time constants of about 2–4 s are expected for the relaxation process of hydrogels with size (radius typically 50 µm) as used here [55,66]. Considering the limitations of the recording time of the instrument, equilibrium appears not to be fully reached where aptamer folding is involved. Therefore, all the presented figures for optical change of the sensor as a function of the K^+^ ion are in semi-equilibrium. The kinetics of the swelling re-equilibration associated with stepwise changes in the Na^+^, Mg^2^^+^ and Ca^2^^+^ concentrations appear to be accounted for by a one component relaxation process and is faster than observed for K^+^.

**Figure 6:**
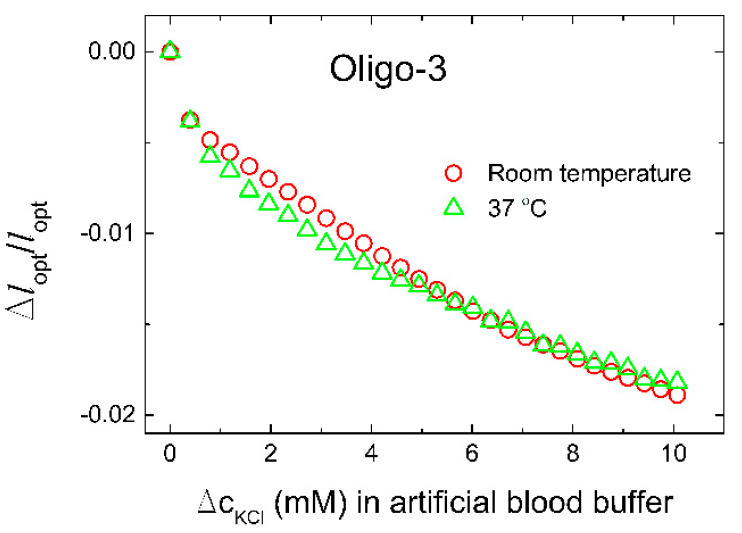
Change in the optical length of the sensor in artificial blood fluid (Tris-HCl with 140 mM NaCl, 2.35 mM CaCl_2_, 0.85 mM MgCl_2_, 42.5 g/L BSA) at room temperature and human body temperature (37 °C).

In order to test whether the designed biosensor has good selectivity, we introduced Na^+^, Tris-HCl buffer (50 mM, pH 7.4, 140 mM NaCl), as the most common potential interfering ions with this type of aptamer. As presented in Figure 4, the influence is negligible. We therefore proceed by evaluation of the selectivity of the oligo-3-co-AAm sensing element in a solution closer to an application by using an artificial blood buffer at room temperature and temperature of the human body (37 °C). Results showed negligible influence of the temperature on the biosensor performance (Figure 6). The sensor yielded a relative deswelling signal up to −2% at 10 mM K^+^ added to the artificial blood buffer, which is significantly less than the K^+^ sensitivity recorded in Tris-HCl buffer (50 mM, pH 7.4, 140 mM NaCl). Possible factors that may contribute to such a difference in sensitivity is that presence of BSA in the artificial blood buffer may affect the level of accessible K^+^ for the oligo-3 aptamer. In this perspective, it is reported that ions interact with proteins, including transport proteins like albumin, but the proteins generally interact stronger with Na^+^ than K^+^. The sensor is tested in environmental conditions of the human blood serum due to the clinical relevance for ICU patients. Furthermore, the sensor could be used in other mediums such as urine, sweat, or wherever potassium tracking is needed. A further perspective is the need for calibration of the actual sensor before a possible development line towards a clinical proof of concept. In this regard, the relative deswelling signal and its uncertainties for the potassium selective response of the oligo-3-co-AAm sensor in artificial blood buffer can be used to provide a limit of detection (LoD). The standard deviations of the Δ*l*_opt_/*l*_opt_ data in artificial blood buffer are observed to be up to 6 × 10^−5^. Accounting for baseline noise at the same level and using three times standard deviation as basis for estimate of LoD, we obtain a LoD of 0.14 mM K^+^ for this particular sensor when used in artificial blood buffer. For the characterization of the sensor in Tris buffer (Figure 4), where the response is larger at the same concentration of K^+^, the LoD will be less, typically half the value of that stated for the artificial blood buffer. These estimates indicate that the sensor is capable to provide good resolution in the determination of K^+^, but one should also be aware that further optimization of the hydrogel material with respect to composition (e.g., the ratio between K^+^ aptamer-supported crosslinks and covalent ones) similar to that reported in previously [39], can be possible.

Note that the present approach allows selective K^+^ determination both in the presence of salt at a level comparable to physiological concentrations, and also in the presence of high protein concentrations. The studies exploiting crown ethers as the moiety hosting cation specificity reports on the K^+^ selectivity not in the presence of a background of other salts, nor in the presence of proteins mimicking physiological conditions more closely. Moreover, the re-equilibration swelling time for the aptamer functionalized hydrogels is found to be much shorter (Figure 5) than the 30 s response time reported for the crown-ether-based selective K^+^ binding [44].

## 4. Conclusions

Herein, the novel aptamer-based biosensor for continuous and label-free in situ potassium monitoring with high sensitivity and selectivity is presented. The influence of Na^+^, Mg^2^^+^, and Ca^2^^+^ ions that could usually affect this type of aptamer is negligible in the solution with ion composition corresponding to human blood conditions. The fabricated aptasensor showed great potential for continuous potassium detection. Moreover, it was tested in artificial blood buffer. Although further testing in human serum and on animals and humans is needed to more fully assess the clinical potential, the data provided here lays the foundation for the design and fabrication of a biosensor for continuous potassium monitoring in the field of medical detection.

## Figures and Tables

**Table 1:** DNA oligonucleotide sequences used for sensors fabrication. Parts of the sequences with aptamer functionality are indicated by bold letters.

Aptamer	Oligonucleotide Sequences
Oligo-3	5′- /Acryd/AAA A**TG AGG GAG GGG** -3′
OligoN	5′- /Acryd/AAA ATG GAC AAA CGA -3′
KLM	5′- /Acryd/AAA A**GG GTT AGG GTT AGG GTT AGG G**AA AAG CGT CCT CCG -3′
L	5′- /Acryd/AAA ACG GAG GAC GC -3′
KLMss	5′- /Acryd/AAA A**GG GTT AGG GTT AGG GTT AGG G** -3′

**Table 2:** Normal and average serum concentrations of the main ionic and protein constituents of human blood.

Component	Normal Range	Average Level
NaCl	135–145 mM	140 mM
KCl	3.60–5.20 mM	4.40 mM
CaCl_2_	2.20–2.50 mM	2.35 mM
MgCl_2_	0.70–1.00 mM	0.85 mM
Albumin	3.50–5.00 g/dL	4.25 g/dL

## Data Availability

Relevant data is included in the manuscript.
